# Responsive immunization and intervention for infectious diseases in social networks

**DOI:** 10.1063/1.4872177

**Published:** 2014-04-24

**Authors:** Qingchu Wu, Haifeng Zhang, Guanghong Zeng

**Affiliations:** 1 College of Mathematics and Information Science, Jiangxi Normal University, Nanchang 330022, China; 2 School of Mathematical Science, Anhui University, Hefei 230039, China

## Abstract

By using the microscopic Markov-chain approximation approach, we investigate the epidemic spreading and the responsive immunization in social networks. It is assumed that individual vaccination behavior depends on the local information of an epidemic. Our results suggest that the responsive immunization has negligible impact on the epidemic threshold and the critical value of initial epidemic outbreak, but it can effectively inhibit the outbreak of epidemic. We also analyze the influence of the intervention on the disease dynamics, where the vaccination is available only to those individuals whose number of neighbors is greater than a certain value. Simulation analysis implies that the intervention strategy can effectively reduce the vaccine use under the epidemic control.

It is well known that the vaccination is very helpful in controlling vaccine preventable disease. When the voluntary vaccination can eradicate the epidemic transmission eventually, two relevant problems are presented: (i) whether it is able to decrease the possibility of epidemic outbreak? (ii) how the vaccine should be used at minimum to yield better result? In this work, we attempt to solve them to some extent. By introducing the responsive immunization based on the local information, we study the impact of the voluntary vaccination on the epidemic threshold. Theoretical analysis and simulation shows that the responsive immunization cannot significantly affect the condition of epidemic. We further analyze the intervention strategy based on the targeted immunization and find that it can effectively reduce the vaccine use. These results may allow to gain new insight into the role of the voluntary vaccination in the epidemic control.

## I. INTRODUCTION

The spread of an epidemic disease (e.g., tuberculosis (TB),^1^ severe acute respiratory syndrome (SARS),^2^ Asian-influenza,^3^ swine-origin influenza A (H1N1)^4,5^) in a population can be studied by using dynamical system approaches. The susceptible-infected-susceptible (SIS) model and the susceptible-infected-recovered/removed (SIR) model are two widely considered models. The theory of complex network can provide an analytic framework for heterogenous contact patterns of a population.^6^ The heterogeneous contact reflects the property that the node degree *k*, or, the number of contacts with other individuals for a given individual, is not uniform. The frequently used network models are Erdös-Rényi (ER) random graphes,^7^ Strogatz-Watts (SW) small-world networks,^8^ and Barabási-Albert (BA) scale-free networks.^9^ There is a sharp contrast in their degree distributions *P*(*k*). The study of epidemic spreading on complex networks shows the existence of a high correlation between the condition of epidemic outbreak and the degree distribution.^10–14^

How to find optimal immunization strategies to minimize the risk of epidemic outbreaks on complex networks have been widely studied.^14–21^ A number of basic immunization strategies have been proposed and investigated, such as the random immunization,^14^ the targeted immunization^14^ and the acquaintance immunization.^15^ These proposed immunization strategies, however, are built on a major premise that vaccination or immunization is compulsory and have not considered the willingness or desire of individuals. Given some social factors, such as, religious belief and human rights, thus, the immunization behavior is not a compulsory behavior but decided by individuals themselves. In this situation, whether to vaccinate or not is related to the risk of being infected by the infectious disease.^18,20^ As a kind of individual awareness,^22,23^ risk assessment of infection should be closely related to an individual's local information (the state of an individual's neighborhood). Hence, individual vaccination behavior depends on its local information. For the convenience, we call such dynamic immunization as the *responsive immunization*,^21^ which is also referred as the information-driven vaccination^19^ or the information dependent vaccination.^24^

In our work^22^ and further work,^25^ the local information affects individual susceptibility and can change the epidemic threshold. An interesting problem is: can the responsive immunization based on the local information affect the epidemic threshold? Intuitively, the answer seems to be “yes.” However, we will find that it is not the case for the responsive immunization. In this paper, we mainly focus on the influence of the responsive immunization on the “epidemic threshold.” Herein, the “epidemic threshold” means the critical value that can discriminate the dynamical behaviors of the system.^26^

The rest of this paper is organized as follows: in Sec. II, an SIS epidemic model with responsive immunization strategy is proposed and the theoretical analysis on the thresholds of the model is given based on the microscopic Markov-chain method. Numerical simulations are also presented to verify the theoretical results. In Sec. III, the external intervention in the responsive immunization is further investigated. At last, conclusions and some discussions are summarized in Sec. IV.

## II. THE SIS MODEL WITH RESPONSIVE IMMUNIZATION

### A. The model

We use the SIS model to investigate the effect of responsive immunization. The SIS model is chosen for the following two reasons: (1) it is widely applicable and may be adapted for some epidemic diseases such as meningitis and gonorrhea;^27^ (2) it is also relatively simple and approximated to the early stage of the epidemic outbreak. We also assume an epidemic spreads along the static network *G* with size *N*, which is completely determined by its adjacency matrix *A* where the entries $a_{ij}=a_{ji}=1$ if there is a link between nodes *i* and *j*, otherwise $a_{ij}=a_{ji}=0$. All nodes of *G* are enumerated with index i=1,2,⋯,N.

In this model with the responsive immunization considered in the SIS model, each individual may stay in one of three states: S-susceptible, I-infected, and V-vaccinated. During a time step, a susceptible individual may get infected at an average rate β per unit time if it is contacted by one infected individual, and *meanwhile* may also be vaccinated and then removed (due to the responsive immunization) at rate $\pi_{\text{vac}}(i)$. An infected individual may recover and become susceptible again at rate γ per unit time. It is assumed that all these events are independent.

Intuitively, the immunization rate $\pi_{\text{vac}}$ increases with both the local information of an epidemic and the response rate of epidemic risk, δ (0≤δ≤1). Suppose node *i* with degree *k_i_* has $k_{\text{inf}}$ ($k_{\text{inf}}\geq 0$) infected neighbors, then the immunization rate of node *i* is given by

$$\pi_{\text{vac}}(i)=\delta \frac{k_{\text{inf}}}{k_{i}}.$$

When δ=0, the model is just the standard SIS epidemic model.^10^ Similar to the previous work, we define the effective spreading rate λ=β/γ. For the sake of the following analysis, we first present a lemma:

**Lemma 1**: Let $V^{*}$ be a set composed of nodes in a network *G* and $p_{j,t}$ be the probability of node $j\in V^{*}$ to be infected at time *t*, respectively. Then the number of infected nodes in $V^{*}$ is a stochastic variable $\xi \in [0,|V^{*}|]$ ($\left|V^{*}\right|$ denoting the number of the elements in set $V^{*}$) and its expected value satisfies

$$E[\xi ]=\sum_{j\in V^{*}}p_{j,t}.$$

**Proof**: Note that

$$E[\xi ]=\sum_{V_{1}\subset V^{*}}\left[\left|V_{1}|\times \Pi_{j\in V_{1}}p_{j,t}\times \Pi_{j\in V^{*}\setminus V_{1}}(1-p_{j,t}\right)\right].$$

According to the inductive method, we can complete the proof.

### B. The microscopic Markov-chain approximation

Previous literatures have indicated that the microscopic Markov-chain approximation (MMA) approach^28,29^ is an effective method in studying the epidemic spreading in quenched networks (i.e., the adjacency matrix is unchanged in time), including unweighted networks,^26,30^ weighted networks,^29,31^ and even multiplex networks.^32^

Along this way, we denote the probability of node *i* to be infected and to be immunized at time *t*, $p_{i,t}$ and $q_{i,t}$, respectively. During the time interval [t,t+1), the change of $p_{i,t}$ depends on two events: the recovery from state I to state S and the infection from state S to state I. The change of $q_{i,t}$ only depends on the vaccination from state S to state V.

Note that for each susceptible node *i*, there exists two exclusive events per unit time: (i) getting infected at rate $\pi_{\text{inf}}(i)$; (ii) becoming immunized at rate $\pi_{\text{vac}}(i)$. Therefore, with these notations the discrete-time epidemic network model is described by

$$\{\begin{array}{c} p_{i,t+1}=(1-\gamma )p_{i,t}+(1-p_{i,t}-q_{i,t})\pi_{\text{inf}}(i),\\ q_{i,t+1}=q_{i,t}+(1-p_{i,t}-q_{i,t})\pi_{\text{vac}}(i). \end{array}$$ (1)

Now, we establish the specific forms of $\pi_{\text{inf}}(i)$ and $\pi_{\text{vac}}(i)$. Let $\zeta_{i,t}$ denote the probability of node *i* being uninfected at time *t*. Considering node *i* may get infected through connections with each of its infected neighbors, the expression of uninfected probability reads^28,29^

$$\zeta_{i,t}=\prod_{j\in N_{i}}\left(1-\beta p_{j,t}\right).$$ (2)

Here, $N_{i}$ denotes the neighborhood of node *i*. Accordingly, we have

$$\pi_{\text{inf}}(i)=1-\zeta_{i,t}=1-\prod_{j\in N_{i}}\left(1-\beta p_{j,t}\right).$$ (3)

We continue to give the expression for $\pi_{\text{vac}}(i)$. Following its definition, we consider the stochastic variable $\xi =k_{\text{inf}}$ ($\xi \leq k_{i}$). By using Lemma 1, we take the expected value of ξ as an approximation to $k_{\text{inf}}$ and obtain

$$\pi_{\text{vac}}(i)≅E[\delta \frac{\xi }{k_{i}}]=\delta \frac{E[\xi ]}{k_{i}}=\delta k_{i}^{-1}\sum_{j\in N_{i}}p_{j,t}.$$ (4)

So Eq. (1) can be rewritten as

$$p_{i,t+1}=(1-\gamma )p_{i,t}+(1-p_{i,t}-q_{i,t})[1-\prod_{j\in N_{i}}\left(1-\beta p_{j,t}\right)]$$ (5a)

$$q_{i,t+1}=q_{i,t}+\delta k_{i}^{-1}(1-p_{i,t}-q_{i,t})\sum_{j\in N_{i}}p_{j,t}.$$ (5b)

When δ=0 and $q_{i,0}=0$ for each *i*, model (5) reduces into the following simple form:

$$p_{i,t+1}=(1-\gamma )p_{i,t}+(1-p_{i,t})[1-\prod_{j\in N_{i}}\left(1-\beta p_{j,t}\right)].$$

This is just the standard SIS model without reinfection terms.^28,29^ According to the previous literature, the critical value of epidemic outbreak for the spreading rate, $\lambda_{c}^{SIS}$, obeys

$$\lambda_{c}^{SIS}=\frac{1}{\Lambda_{\text{max}}(A)},$$ (6)

where $\Lambda_{\text{max}}(A)$ is the leading eigenvalue of the adjacency matrix A. When the spreading rate is larger than the critical value, the epidemic disease will become endemic and persist in a population.

### C. The steady state and the critical value of epidemic spreading

For the approximation model (5), we first analyze the steady state of its dynamical behavior. To this end, we would like to determine the values of $p_{i,t}$ and $q_{i,t}$ at the steady state, *p_i_* and *q_i_*. On substituting $p_{i,t}\equiv p_{i}$ and $q_{i,t}\equiv q_{i}$ into Eqs. (5), we have

$$\{\begin{array}{c} p_{i}=(1-\gamma )p_{i}+(1-p_{i}-q_{i})[1-\prod_{j\in N_{i}}\left(1-\beta p_{j}\right)],\\ q_{i}=q_{i}+\delta k_{i}^{-1}(1-p_{i}-q_{i})\sum_{j\in N_{i}}p_{j}. \end{array}$$ (7)

As long as δ>0, we have $(1-p_{i}-q_{i})\sum_{j\in N_{i}}p_{j}=0$. This indicates two possible cases: (i) $\sum_{j\in N_{i}}p_{j}=0$; (ii) $1-p_{i}-q_{i}=0$. When $\sum_{j\in N_{i}}p_{j}=0$, we have $p_{j}=0$ for each $j\in N_{i}$ and further $1-\prod_{j\in N_{i}}\left(1-\beta p_{j}\right)=0$. In either case, $(1-p_{i}-q_{i})[1-\prod_{j\in N_{i}}\left(1-\beta p_{j}\right)]\equiv 0$. This means that for each node *i*, $p_{i}=0$ but the value of *q_i_* is unknown and related to the initial conditions of the system. Therefore, the fraction of infected node in a population (denoted by *I*(*t*)) always decays to zero regardless of the spreading rate λ and the response rate δ (δ>0).

Next, we want to estimate the critical value of epidemic spreading for our model, that is, the epidemic threshold $\lambda_{c}$. Based on the analysis of the steady state, the epidemic threshold means: if $\lambda \leq \lambda_{c}$, *I*(*t*) decreases to zero (no epidemic), otherwise, first increases to a maximum and then decreases to zero (an epidemic) due to the responsive immunization. Following,^33,34^ the occurrence or not of an epidemic depends on the stability of the disease free equilibrium of the disease model described by (5).

Additionally, we notice that $I(t)=(1/N)\sum_{i=1}^{N}p_{i,t}$. Therefore, in order to obtain the mathematical expression of the epidemic threshold we only consider the subsystem (5a) near the disease free equilibrium ($p_{i}=0$ and $0\leq q_{i}\leq 1$ for each *i*). At this time, a linear form of Eqs. (5a) can be written as

$$p_{i,t+1}=(1-\gamma )p_{i,t}+\beta (1-q_{i})\sum_{j=1}^{N}a_{ij}p_{j,t}.$$ (8)

This uses the approximation (1−a)(1−b)≃1−a−b when a≪1,b≪1.

This system is not a closed form since we do not know *q_i_* value corresponding to $p_{i}=0$ for each *i*. However, we can approximately analyze the stability of the disease free equilibrium under the assumption (H): $p_{i,0}≃0$ and $q_{i,0}=0$ for each *i*. In fact, from Eqs. (5), when $p_{i,0}=0$ we have $p_{i,t}=0$ for t>0 and then $q_{i,t}=0$. In other words, under the assumption (H) $p_{i,0}\to 0$ can imply that $q_{i,t}\to 0$ and further $q_{i}\to 0$. Hence, we consider the system near the zero solution ($p_{i}=q_{i}=0$ for each *i*). At this time, model (8) becomes

$$p_{i,t+1}=(1-\gamma )p_{i,t}+\beta \sum_{j=1}^{N}a_{ij}p_{j,t}.$$ (9)

Now we study the stability of the above system. Let us introduce a vector function $p_{t}={\left(p_{1,t},p_{2,t},...,p_{N,t}\right)}^{T}\in R^{N}$ (the state vector of the network). Then, Eq. (9) can be given by a collective form

$$p_{t+1}=(1-\gamma )p_{t}+\beta Ap_{t}=[\beta A+(1-\gamma )I]p_{t}.$$ (10)

Here, I denotes a N-dimensional identity matrix. From above, the local stability of the zero solution of system (10) can be established by

$$\Lambda_{\text{max}}[\beta A+(1-\gamma )I]<1.$$

This indicates that the epidemic threshold for the spreading rate, $\lambda_{c}$, is given by

$$\lambda_{c}=\frac{1}{\Lambda_{\text{max}}(A)}.$$ (11)

The threshold condition is the same as that in the standard SIS model (6) and indicates that the response rate δ has no impact on the epidemic threshold. This differs significantly from our previous work without vaccination,^22^ where we have shown that the local information can affect the epidemic threshold. We argue that the epidemic threshold is unchanged for different δ values because: (1) the response rate does not directly affect the dynamic of infection but decrease the number of susceptible nodes; (2) at the beginning of an epidemic spreading, the vaccination fraction generated by the responsive immunization is not large enough to halt the epidemic outbreak.

It is worth stressed that the analysis of the epidemic threshold is on the basis of the assumption (H). If the assumption is not satisfied, the epidemic threshold should be related with initial conditions (i.e., $p_{i,0}$, $q_{i,0}$). In addition, considering the effectiveness of the MMA approach, we believe that the responsive immunization has no significantly affect on the epidemic threshold in the stochastic model, together with the following simulation analysis.

### D. Simulations

To test above argument, we perform Monte Carlo simulations over both BA scale-free networks^9^ with the degree distribution $P(k)∼k^{-3}$ and ER networks with connecting probability p=0.006.^7^ The Monte Carlo simulations are implemented in a parallel way, that is, each node's state can be updated with a certain rate in a time step. More specifically, for a susceptible node *i* at each time step, we generate a random number r∈[0,1), if $r\in [0,\pi_{\text{inf}}(i))$ then node *i* is changed from state S to state I; else if $r\in [\pi_{\text{inf}}(i),\pi_{\text{inf}}(i)+\pi_{\text{vac}}(i))$ then node *i* is changed from state S to state V; else node *i* is still susceptible. Here, $\pi_{\text{inf}}(i)$ and $\pi_{\text{vac}}(i)$ are the transition rates as stated above. One should note that, since we mainly focus on the epidemic threshold of the model, near this critical point, the epidemic just begins to prevail, so the infected neighbors are few, in this situation, we can image that the value of $\pi_{\text{inf}}(i)+\pi_{\text{vac}}(i)$ is smaller than 1. Although the network considered here is small (*N* = 2000), we also made simulations for a larger network (e.g., *N* = 5000) and obtained a similar observation.

We first verify the accuracy of Eq. (11). Simulations begin with a single seed initial condition. To minimize random fluctuation caused by the initial conditions, we make average over 200 realizations of different initial infected nodes.

**FIG. 1.**
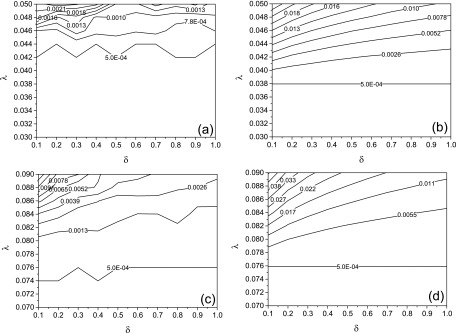
The contour plot of the maximal infection density $I_{\text{max}}$, where the x coordinate is the response rate δ and the y coordinate is the spreading rate λ on a BA network with the mean degree ⟨k⟩≅12 (a) (b) and an ER network with p=0.006 (c) (d). Plots (a) and (c) denote the Monte Carlo simulations and plots (b) and (d) represent the Markov chain predictions (5). In all simulations the recover rate γ=1. The bottom line in each plot forms the boundary between the epidemic outbreak and the epidemic extinction.

In Fig. 1, we compare the Monte Carlo simulations and theoretical model (5) on the epidemic threshold, $\lambda_{c}$. The Markov chain prediction shows that the epidemic threshold is indeed independent of the parameter δ, which complies with the analysis from Eq. (11). In order to further verify this formula, we compute the value of $\Lambda_{\text{max}}(A)$ for the BA scale-free network used here according to the power method and obtain that $\Lambda_{\text{max}}(A)≃26.325$. Following Eq. (11), we have that $\lambda_{c}=1-\Lambda_{\text{max}}(A)≃0.03799$, which is a good approximation to the simulation results obtained from Eqs. (5) (Fig. 1(b)). For an ER random network, $\Lambda_{\text{max}}(A)≃13.202$, so $\lambda_{c}=1-\Lambda_{\text{max}}(A)≃0.07575$. This also complies with the simulation results obtained from Eq. (5) (Fig. 1(d)).

The epidemic threshold for the Monte Carlo simulation on a BA scale-free network (Fig. 1(a)) is larger than that for the Markov chain prediction based on Eqs. (5) (Fig. 1(b)). This is mainly due to the first order approximation of the mathematic model.^35^

Hinted by the real situations in which the responsive immunization often takes place after the beginning of the outbreak of the epidemic (for an emerging infectious disease at least), we consider another kind of initial condition: a portion of infected seeds, e.g., 1% of the nodes are infected. Unless otherwise specified, we set the recovery rate γ=0.5 in the later simulations.

**FIG. 2.**
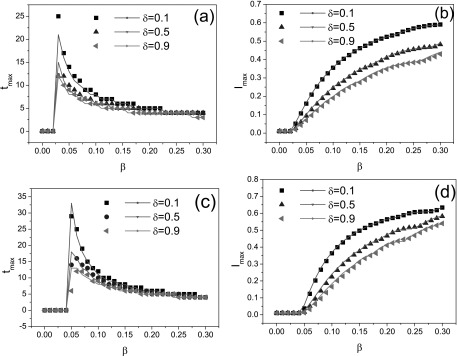
Illustrations of the peak time $t_{\text{max}}$ for a BA network (a) and an ER network (c), the maximal infection density $I_{\text{max}}$ for a BA network (b) and an ER network (d) as functions of the infection rate β. The solid lines denote the theoretical predictions. In four figures, three curves correspond to different response rates. From top to bottom: δ=0.1,0.5, and 0.9. All simulation results are averaged over 200 epidemic dynamics.

Herein, we would like to examine the exactness of the theoretical model (5) compared to the Monte Carlo simulation. In order to do this, we consider the maximal infection density $I_{\text{max}}$ and the corresponding peak time $t_{\text{max}}$ over different BA scale-free networks and ER random networks with the almost same mean degree, respectively. Fig. 2 shows the peak time and the maximal infection density as functions of the infection rate for different δ values, which are consistent with theoretical models as indicated by the solid lines. This figure tells us that the MMA approach is effective to model the epidemic spreading with responsive immunization, even for the prediction of the peak time.

Furthermore, Fig. 2 suggests that, though the epidemic threshold is not related to the value of δ, increasing the value of δ can lower the peak time $t_{\text{max}}$, indicating the upward tendency of epidemic is fast controlled. Meanwhile, the maximal infection density $I_{\text{max}}$ also decreases with the value of δ, which means that the responsive immunization can effectively control or hinder the prevalence of epidemic.

In the final part of this section, we investigate the *initial* outbreak of epidemic, which means that *I*(*t*) increases at *t* = 0. For the discrete-time system (5), it is needed to consider the quantity

$$\Delta I=I(1)-I(0)=\frac{\sum_{j}p_{j,1}-\sum_{j}p_{j,0}}{N}.$$ (12)

Considering the average over initial conditions, we have $p_{i,0}=\varepsilon$ and $q_{i,0}=0$ for each *i*. Then,

$$\begin{array}{c} \sum_{j}p_{j,1}-\sum_{j}p_{j,0}=-\gamma \varepsilon N+(1-\varepsilon )\sum_{i}\left[1-{\left(1-\beta \varepsilon \right)}^{k_{i}}\right]\\ ≃-\gamma \varepsilon N+\beta \varepsilon \sum_{i}k_{i}\\ ≃\left(-\gamma +\beta \langle k\rangle \right)\varepsilon N. \end{array}$$

On plugging the above equality into Eq. (12), we obtain

ΔI≃−γε+ε⟨k⟩β.

From this equality, the critical value of *initial* epidemic outbreak obeys

$$\lambda_{c}^{0}=\frac{1}{\left\langle k\right\rangle }.$$ (13)

Interestingly, we find that $\lambda_{c}^{0}$ is inversely proportional to the mean degree of the network. When $\lambda >\lambda_{c}^{0}$, ΔI>0 and *I*(*t*) first increases; when $\lambda <\lambda_{c}^{0}$, we have ΔI<0 and the epidemic prevalence first decreases. One should note that, this condition is used to judge whether the epidemic will outbreak at *initial* time step, though $\lambda <\lambda_{c}^{0}$, the outbreak of epidemic is also possible to happen since $\lambda_{c}^{0}>\lambda_{c}$ (as illustrated in the latter simulation).

**FIG. 3.**
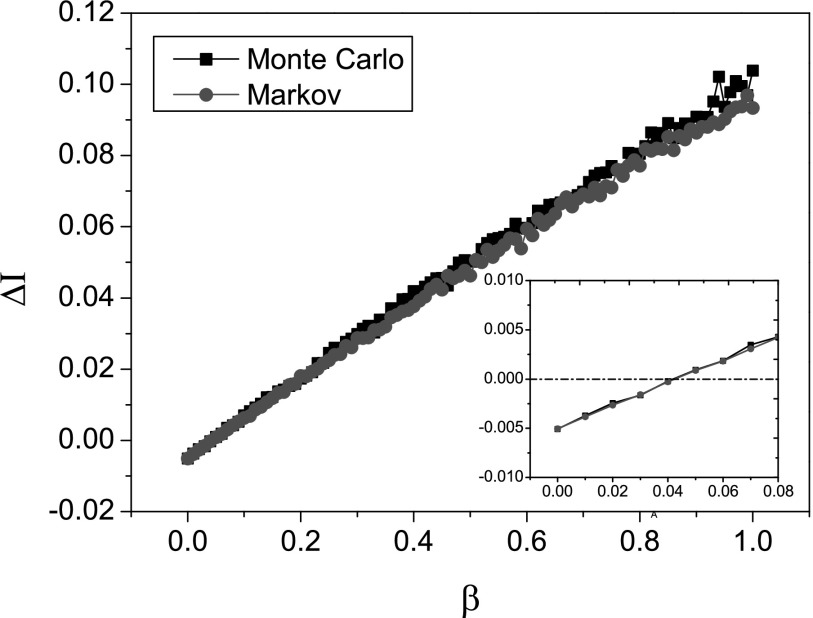
Initial change ΔI as a function of the infection rate β when δ=0.5 and ⟨k⟩≅12. The inset shows the zoom in results for the range β∈[0,0.08]. The simulation result is averaged over 200 epidemic dynamics.

In Fig. 3, we illustrate the change of ΔI on a BA scale-free network with the mean degree ⟨k⟩≅12. Note that ε=0.01 and γ=0.5, so we have ΔI=−0.005+0.12β. This tells us that ΔI is a linear function of the infection rate β, which can be seen in this figure. Also, we can derive the condition of initial epidemic outbreak: $\lambda >\lambda_{c}^{0}\Rightarrow \beta >0.0417$. In the inset of Fig. 3, we can see that ΔI>0 only if β≥0.05, which is in accordance with the Markov chain prediction.

## III. INTERVENTION IN THE RESPONSIVE IMMUNIZATION

According to the content of the responsive immunization, each individual potentially vaccinates with a certain rate. Hence, the vaccine coverage may range from nodes with small degree to ones with large degree. In order to investigate the range of the vaccine coverage in our model, we consider the degree distribution of vaccinated nodes (called as the *vaccination degree distribution*)

$$F(k)=\frac{number\;of\;vaccinated\;nodes\;with\;degree\;k}{total\;number\;of\;vaccinated\;nodes},$$

which can be compared to the degree distribution of all nodes in the network *P*(*k*) by using numerical simulations.

**FIG. 4.**
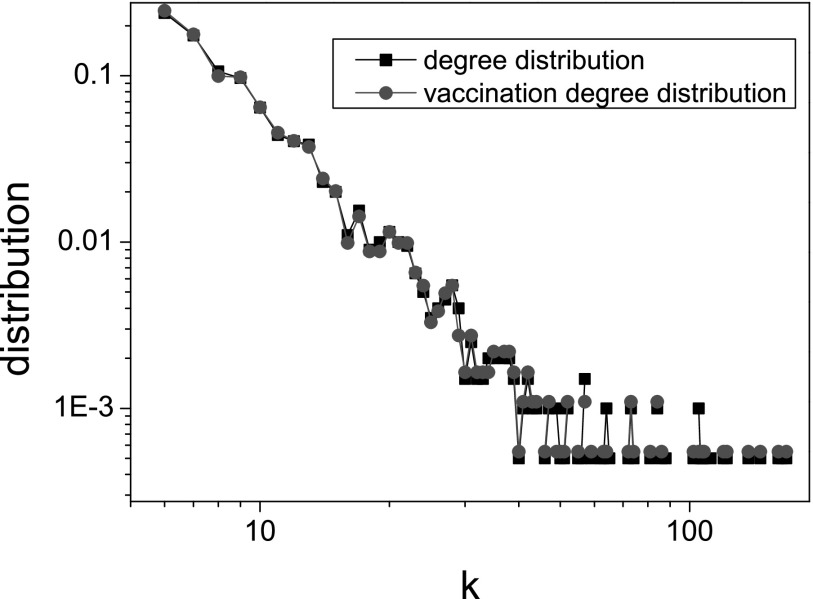
The degree distribution of all nodes and the vaccination degree distribution of all vaccinated nodes in a BA scale-free network with ⟨k⟩≅12. Other parameters: δ=0.1,γ=0.5, and β=0.2.

**FIG. 5.**
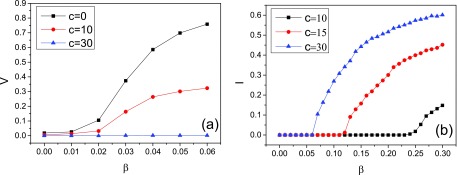
(a) The fraction of vaccinated nodes at the steady state is shown as a function of the infection rat β. This figure allows us to compare different intervention levels about the vaccination sizes. (b) The fraction of infected nodes at the steady state is shown as a function of the infection rate β. This figure indicates another epidemic threshold above which an epidemic can undergo a new outbreak and persist in a population. Parameters δ=1 and γ=0.5. Numerical simulation results are carried out and averaged over 100 epidemic dynamics.

In this section, we use BA scale-free networks to simulate epidemic dynamics and initially 1% of the nodes are infected. As shown in Fig. 4, the relation between two kinds of degree distribution is $F(k)∼P(k)∼k^{-3}$. The good relation should be induced by the degree uncorrelated property of BA networks and it would be interesting to consider the impact of network structure on the vaccination degree distribution and its relation with the degree distribution in other work. Nevertheless, this tells us that the vaccination possibility of the node with small degree is nearly equal to that of the node with large degree, which forces a large amount of vaccines to be required for the responsive immunization (see Fig. 5(a): c = 0). However, the situation that too many people choose vaccination potentially leads to the waste of resources. Especially when the vaccine is rare, it may cause social panic, even violence. So we should take some necessary intervention to the voluntary vaccination.

It is well known that the targeted immunization scheme in scale-free networks is very effective in controlling the epidemic outbreak.^14^ Inspired by this, we introduce the intervention measure based on the targeted immunization and only permit those nodes with large degree to take vaccine—when node *i* goes to the epidemic prevention station to vaccinate, we only allow those nodes with degree $k_{i}\geq c$ to get vaccinated. In other words, we modify the above model with responsive immunization as follows:

$$\pi_{\text{vac}}(i)=\{\begin{array}{c} \delta k_{i}^{-1}k_{\text{inf}},\;k_{i}\geq c;\\ 0,\;\text{otherwises}. \end{array}$$ (14)

Here, *c* reflects the level of vaccination intervention. The larger the value of *c*, the higher the intervention level. When *c* = 0, there is no any vaccination intervention. As above analysis, an infectious disease dies out eventually. However, in this case the vaccination fraction is generally large. Hence, we consider the impact of the external intervention (i.e., c>0) on the vaccination fraction.

In Fig. 5(a), we report the change of the vaccination fraction at the steady state $V=V_{\infty }(c)$ for different *c* values when the maximal response rate (i.e., δ=1) is assumed. In this figure, we choose the parameter window β∈[0,0.06] where for all cases become zero/vanish as t→∞ (Fig. 5(b)). This can allow us to clearly observe the intervention effect. As a result, *V* decreases significantly with increasing *c* value. We take the case β=0.05 as an illustration. In this case, $V_{\infty }(0)≅70\%$, while $V_{\infty }(10)≅30\%$ and $V_{\infty }(30)≅1\%$.

For the model with no intervention, the infection density becomes zero eventually. But for the model with degree-based intervention, the change of infection density *I*(*t*) may not be like that. Actually, as shown in Fig. 5(b), when the infection rate is large enough there exists a positive value of infection density at the steady state. This determines another epidemic threshold $\lambda_{c}^{\prime }$. When $\lambda >\lambda_{c}^{\prime }$, $I=I_{\infty }(c)>0$; when $\lambda <\lambda_{c}^{\prime }$, *I* = 0. Therefore, the intervention can lead to another larger epidemic threshold, above which the infectious disease will undergo a new outbreak and persist in the population. This is not desirable from the perspective of epidemic control and can be solved by increasing the vaccine coverage. When the vaccine is rare, we should implement other strategies, e.g., quarantine strategies.

Besides the SIS-like epidemic threshold $\lambda_{c}^{\prime }$, the system has two other critical values: (1) the threshold of initial epidemic outbreak $\lambda_{c}^{0}$; (2) the SIR-like epidemic threshold $\lambda_{c}$ above which the epidemic disease asymptomatically decays. We can approximately evaluate these quantities. Actually, after substituting the modified form of $\pi_{\text{vac}}(i)$ (14) into model (1) and using the similar analysis, we obtain that $\lambda_{c}^{0}=1/\langle k\rangle ,$ and $\lambda_{c}≅1/\Lambda_{\text{max}}(A),$ where the usage of the approximation symbol accounts for the impact of a portion of initial infection condition. These equalities indicate that the two parameters δ and *c* almost have no impact on both $\lambda_{c}^{0}$ and $\lambda_{c}$.

Since $\Lambda_{\text{max}}(A)$ is never smaller than the mean degree of the network, ⟨k⟩, $\lambda_{c}\leq \lambda_{c}^{0}$.^30^ What is more, $\lambda_{c}$ must be less than $\lambda_{c}^{\prime }$. When $\lambda_{c}^{\prime }\neq \lambda_{c}^{0}$, the dynamical behavior of the system under consideration is completely characterized by three critical values of spreading rate ($\lambda_{c},\lambda_{c}^{0}$, and $\lambda_{c}^{\prime }$). As an illustration, we consider the intervention model for the case *c* = 30 and γ=0.5 on a BA scale-free network. According to our simulations and computations, three critical values of infection rate $\beta_{c}≃0.02,\beta_{c}^{0}≃0.04$, and $\beta_{c}^{\prime }≃0.06$. Therefore,

$$\beta_{c}<\beta_{c}^{0}<\beta_{c}^{\prime }.$$

**FIG. 6.**
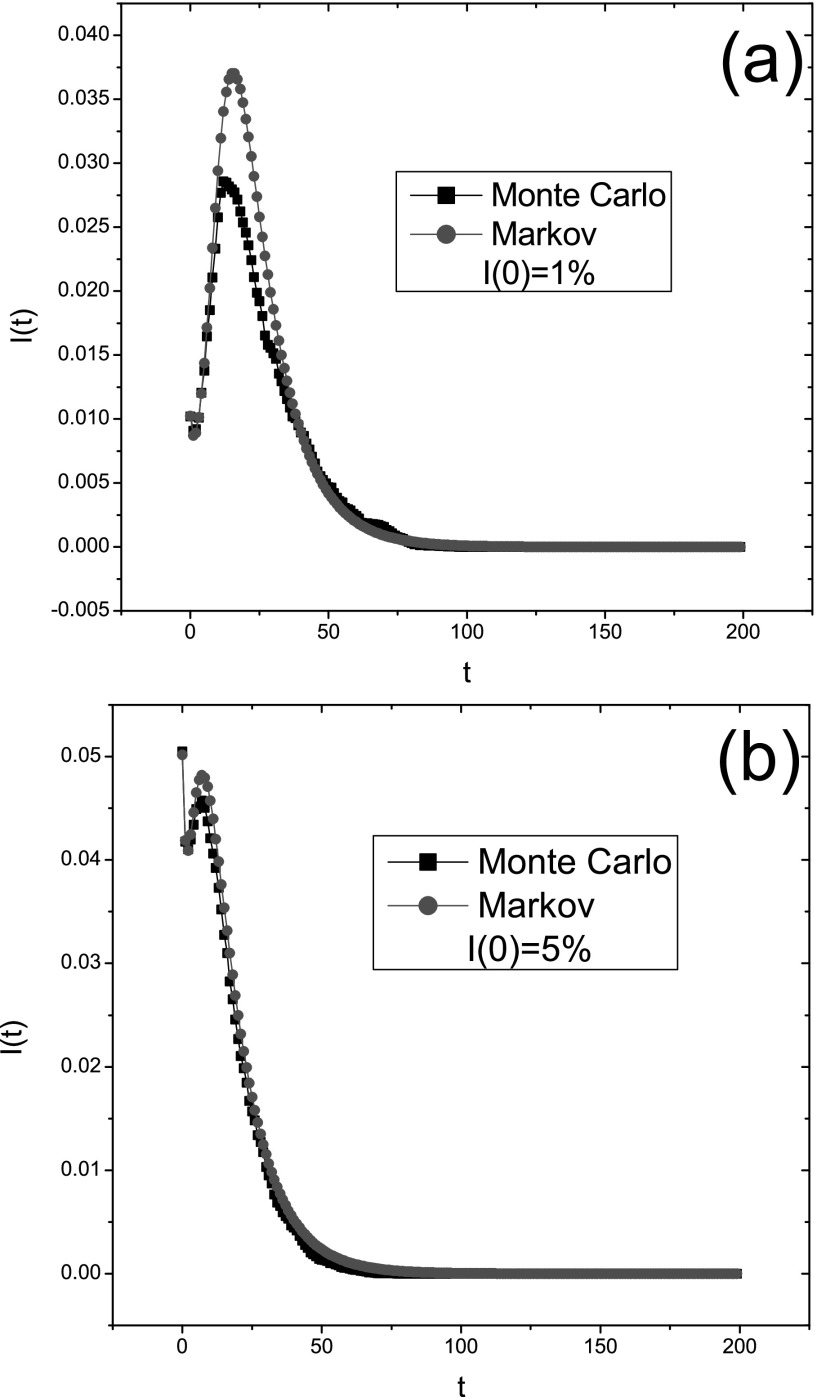
The fraction of infected nodes in a population *I*(*t*) as a function of time in the same BA scale-free network with *N* = 2000 for different initial infection conditions: (a) I(0)=1%; (b) I(0)=5%. The simulation is obtained by taking the average over 100 epidemic dynamics. Other parameters: δ=1,γ=0.5,β=0.03, and *c* = 30. This figure shows that the deviation between them can be reduced by increasing the initial infection density.

When the infection rate lies between $\beta_{c}$ and $\beta_{c}^{0}$, the epidemic prevalence first decreases, then increases, and finally drops to zero. Fig. 6 shows that the change of the infection curve *I*(*t*) is just like that. We also notice the deviation at the peak time between the Monte Carlo simulation and the Markov chain prediction. This may be related to the number of infected seeds distributed in the network initially. When this number is very small, a possible outbreak may be eradicated due to the simulation randomness. Hence, we can see that the deviation becomes smaller with increasing *I*(0) values as illustrated in Fig. 6(b).

## IV. CONCLUSIONS AND DISCUSSIONS

As a brief summary, a modified SIS epidemic model with the responsive immunization is proposed and analyzed. Our main results are: (1) we derive the epidemic threshold of the model and find that $\lambda_{c}=1/\Lambda_{\text{max}}(A)$; (2) we establish the critical condition of initial epidemic outbreak; (3) we obtain the impact of the intervention on the epidemic dynamics and final vaccination size.

Recently, Sahneh and *et al.*^26^ found two kinds of epidemic thresholds that can discriminate the asymptotical decay and the exponential decay of an epidemic disease in a susceptible-alert-infected-susceptible (SAIS) model. In our intervention model, we also find the existence of multiple critical values of spreading rate. Interestingly, the meanings of $\lambda_{c}$ and $\lambda_{c}^{\prime }$ are just as stated in Ref. 26. From the viewpoint of epidemic control, it is not enough to raise the larger threshold $\lambda_{c}^{\prime }$ because the epidemic still breaks out when the spreading rate λ satisfies $\lambda_{c}<\lambda <\lambda_{c}^{\prime }$.

In our model, the responsive immunization has no significant influence on the epidemic threshold and a large vaccination fraction is required to halt the epidemic spreading. It is necessary to take some intervention measures in the voluntary vaccination. We argue that there are two types of intervention measures according to their effects: (1) one is that the epidemic threshold becomes larger; (2) the other is that the epidemic threshold is unchanged but final vaccination size decreases. In the present paper, the degree-based intervention based on the targeted immunization belongs to the second type since it can only reduce the vaccine size. One may consider the first type of vaccination intervention, e.g., the responsive immunization on the adaptive contact network^17^ where the epidemic threshold may be changed due to the change of network structure.
